# Genome-wide analysis of Dof family transcription factors and their responses to abiotic stresses in Chinese cabbage

**DOI:** 10.1186/s12864-015-1242-9

**Published:** 2015-01-31

**Authors:** Jing Ma, Meng-Yao Li, Feng Wang, Jun Tang, Ai-Sheng Xiong

**Affiliations:** State Key Laboratory of Crop Genetics and Germplasm Enhancement, College of Horticulture, Nanjing Agricultural University, Nanjing, 210095 China

**Keywords:** Dof, Transcription factor, Chromosomal location, Gene duplication, qRT-PCR, Chinese cabbage

## Abstract

**Background:**

Chinese cabbage is an important leaf vegetable that experienced long-term cultivation and artificial selection. Dof (DNA-binding One Zinc Finger) transcription factors, with a highly conserved Dof domain, are members of a major plant-specific transcription factor family that play important roles in many plant biological processes. The Dof family transcription factors, one of the most important families of transcriptional regulators in higher plants, are involved in massive aspects of plant growth, development, and response to abiotic stresses. Our study will supply resources for understanding how Dof transcription factors respond to abiotic stress and the interaction network of these genes in tolerance mechanism.

**Results:**

In this study, we performed a comprehensive analysis of Dof family factors in Chinese cabbage. In total, 76 genes encoding BraDof family transcription factor were identified from Chinese cabbage, and those BraDof factors were divided into nine classes. Fifteen motifs were found based on Dof amino acid sequence alignments. Chromosome locations and gene duplications of *BraDof* family genes were also analyzed. Ten duplicate events of *BraDof* genes were discovered in Chinese cabbage chromosomes. The uneven distribution of *BraDof* genes in *Brassica* chromosomes may cause the expansion of *BraDof* genes. In the Dof family, 37 and 7 orthologous genes were identified between Chinese cabbage and *Arabidopsis* and between Chinese cabbage and *Oryza sativa*, respectively. The interaction networks of *Dof* factors in Chinese cabbage were also constructed. Expression profiles of nine selected genes from different nine classes subjected to four abiotic stresses (cold, heat, salt and drought) were further investigated by quantitative real-time PCR to obtain a better understanding of the functions and regulation mechanisms of BraDof family transcription factors in two Chinese cabbage varieties, ‘Lubaisanhao’ and ‘Qingdao 87-114’.

**Conclusions:**

Dof-family transcription factors were analyzed in genome of Chinese cabbage. Chromosomal locations showed that duplication might result in expansion. Response to abiotic stresses was elucidated in Chinese cabbage varieties. The results provide novel insights into the stress responses of *BraDof* genes and promote a better understanding of the construction and function of Dofs in Chinese cabbage.

**Electronic supplementary material:**

The online version of this article (doi:10.1186/s12864-015-1242-9) contains supplementary material, which is available to authorized users.

## Background

Chinese cabbage (*Brassica rapa* L. ssp. *pekinensis*), an economically important vegetable in Asia, belongs to the family Cruciferae, species *Brassica* [1,2]. ‘Lubaisanhao’ and ‘Qingdao 87-114’ are two important Chinese cabbage varieties that share several identical traits, such as high yield, good quality, and disease resistance. In green-house condition, ‘Lubaisanhao’ grow better than ‘Qingdao 87-114’ in lower temperature condition, however, ‘Qingdao 87-114’ showed better resistance to high temperature in summer. ‘Lubaisanhao’ can be planted in relatively low temperature environments, while ‘Qingdao 87-114’ can grow in Nanjing District, southern China with heat tolerance. ‘Lubaisanhao’ suit cooler and wetter condition during seedling stage, however, ‘Qingdao 87-114’ can fit hotter temperature environment [3].

Plants grow in complicated environments and survive many stresses, such as cold, heat, drought, and soil salinization. These abiotic stresses cause drops in crop production and declines in quality [4,5]. To resist damage, plants must evolve to regulate plant-specific signals by gene expression for specific physiological and metabolic pathways [6,7]. Transcription factors are a special type of regulators with highly conserved specific DBDs (DNA-binding domains) involved in stress resistance [5,8].

Dof (DNA-binding One Zinc Finger) transcription factors, a family of plant-specific transcription factors, play important roles in many fundamental processes in higher plants, such as photosynthesis, stress responses, seed germination, flower induction, and light-mediated circadian rhythms [9-12]. Dof transcription factors generally contain 200–400 amino acids with a highly conserved 52-amino acid Dof domain [10,13]. The Dof domain is located on the N-terminus, which has been identified as a DNA-binding domain, and features the structure of a Cys2/Cys2 Zn^2+^ finger recognizing a *cis*-regulatory element comprising the common core sequence (AT)/AAAG [10,13-17]. The DNA-binding domain is a critical region regarded as a bi-functional domain that combines with DNA and interacts with other proteins [10,14,18]. A transcriptional regulation domain at the C-terminal region may perform diverse functions because of its interactions with different regulatory proteins and activation of gene expression [10,14,19,20].

Many studies have demonstrated that Dof transcription factors are involved in plant growth and development, seed germination, photosynthesis, and biotic/abiotic stress responses in many species, but little is known in Chinese cabbage. Till now, the abiotic stress responses of *BraDof* genes in Chinese cabbage are unknown. The objective of this study was to establish an extensive picture of the BraDof family transcription factors in Chinese cabbage.

In this study, we identified 76 BraDof members in Chinese cabbage based on genome sequences and divided them into nine classes. The proportions of these nine classes of *BraDof* family genes, gene duplications, and chromosomal locations were also investigated for further study. Expression profiles under four stress treatments (cold, heat, salt, and drought) were evaluated to determine the responses of *BraDof* genes to abiotic stresses in two varieties of Chinese cabbage. The results will provide novel insights into the stress responses of *BraDof* genes and promote a better understanding of the construction and function of Dofs in Chinese cabbage.

## Results

### Identification and analysis of BraDof transcription factors

**Figure 1:**
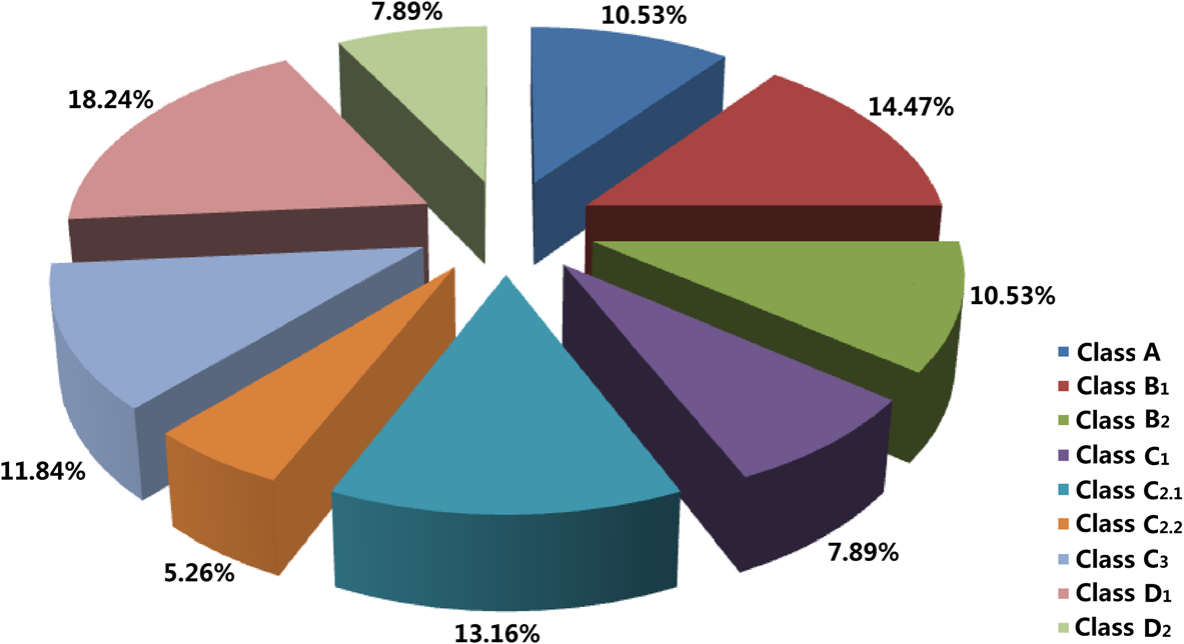
**Proportions of various BraDof classes in Chinese cabbage.** The nine different colors indicate the nine BraDof classes of Chinese cabbage.

To identify and analyze BraDof factors, we identified the BraDof members in Chinese cabbage based on genome sequences. A total of 76 genes encoding BraDof family transcription factors were identified from genome sequence analysis in Chinese cabbage (Additional file 1: Tables S1 to S11). The BraDof family factors were divided into nine classes on the basis of the predicted Dof domains as follows: Classes A, B_1_, B_2_, C_1_, C_2.1_, C_2.2_, C_3_, D_1_, and D_2_. Class D_1_ showed the largest number of BraDof factors (18.42%), whereas Class C_2.2_ showed the least number of BraDof family factors (5.26%) (Figure 1). These different 9 classes (A, B_1_, B_2_, C_1_, C_2.1_, C_2.2_, C_3_, D_1_, and D_2_) represent each sub-family of Dof family transcription factors, and the 9 classes we classified based on the predicted Dof domains analogize by the phylogenetic tree by using 46 *A. thaliana* Dof genes as references [9,10,21] . The proportion of the nine classes is not even. Two sets of classes shared the same proportions: Classes A and B_2_ showed individual proportions of 10.53% (eight members) and Classes C_1_ and D_2_ showed individual proportions of 7.89% (six members) (Figure 1).

### Characterization of the deduced amino acid sequences of BraDofs

Most BraDofs share the similar physical and chemical characteristics, but there were several exceptions. ExPASy, Sequence Manipulation Suite, and RPSP were used to analyze the physical and chemical characteristics of BraDofs from Chinese cabbage; the data are summarized in Additional file 1: Table S12. The theoretical pI of Class B_1_ was about 9, and only one BraDof factor (BraDof028) showed a theoretical pI of 8.77. The results in theoretical pI value were also observed in Class B_2_, however, this class had a pI of 10.14. Classes A and C_2.1_ showed theoretical pI values of 7–10. Class C_2.2_ yielded the lowest theoretical pI of 5. All other classes had complex theoretical pI ranging in value from 5 to 10. The percentage of aliphatic amino acids in all of the BraDofs was slightly higher than the percentage of aromatic amino acids. The percentage of positive amino acids was twice as large as the percentage of negative amino acids. The proportions of aliphatic and positive amino acids in most of the BraDofs were > 10%, contrasting with the proportions of aromatic and negative amino acids, which were < 10%.

Theoretical predictions of protein solubility are important in determining the structure and stability of a protein. In total, the percentage of insoluble recombinant proteins of most of the BraDofs was > 97%. The percentage of insoluble recombinant proteins in all of the BraDofs of Classes B_1_, C_1_, and D_2_ was > 97%. By contrast, the percentage of insoluble recombinant proteins of some BraDofs of Classes A, B_2_, and C_2.1_ was < 97%. Class C_2.2_ showed a percentage of insoluble recombinant proteins relatively lower than those of other classes (<90%). The percentages of insoluble recombinant proteins in Classes C_3_ and D_1_ ranged from 50 to > 97%.

### Motif location and phylogenetic relationship analysis of BraDofs

**Figure 2:**
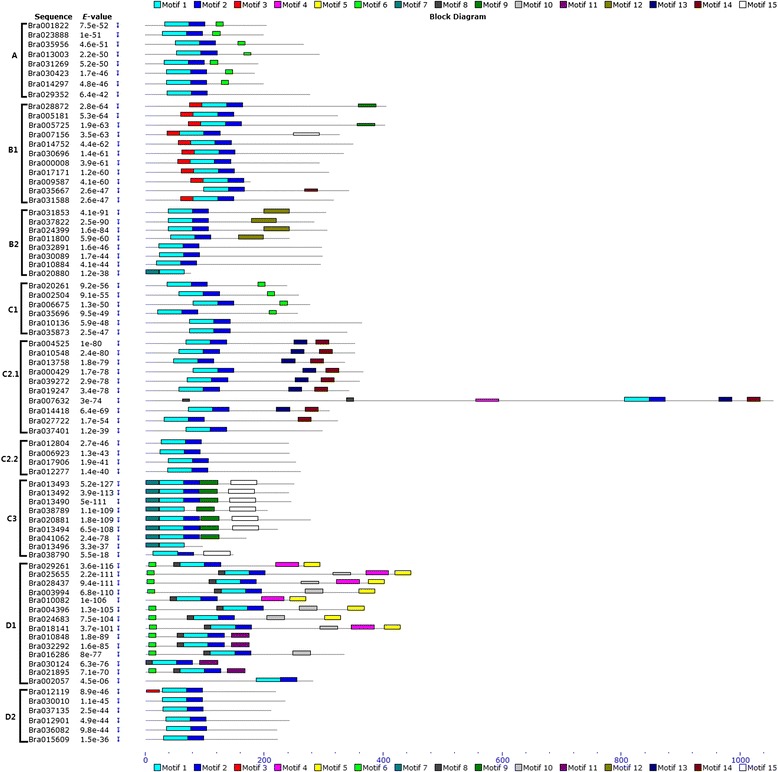
**Common motifs of BraDof family proteins in Chinese cabbage.** Dof domains are represented by striped boxes.

**Figure 3:**
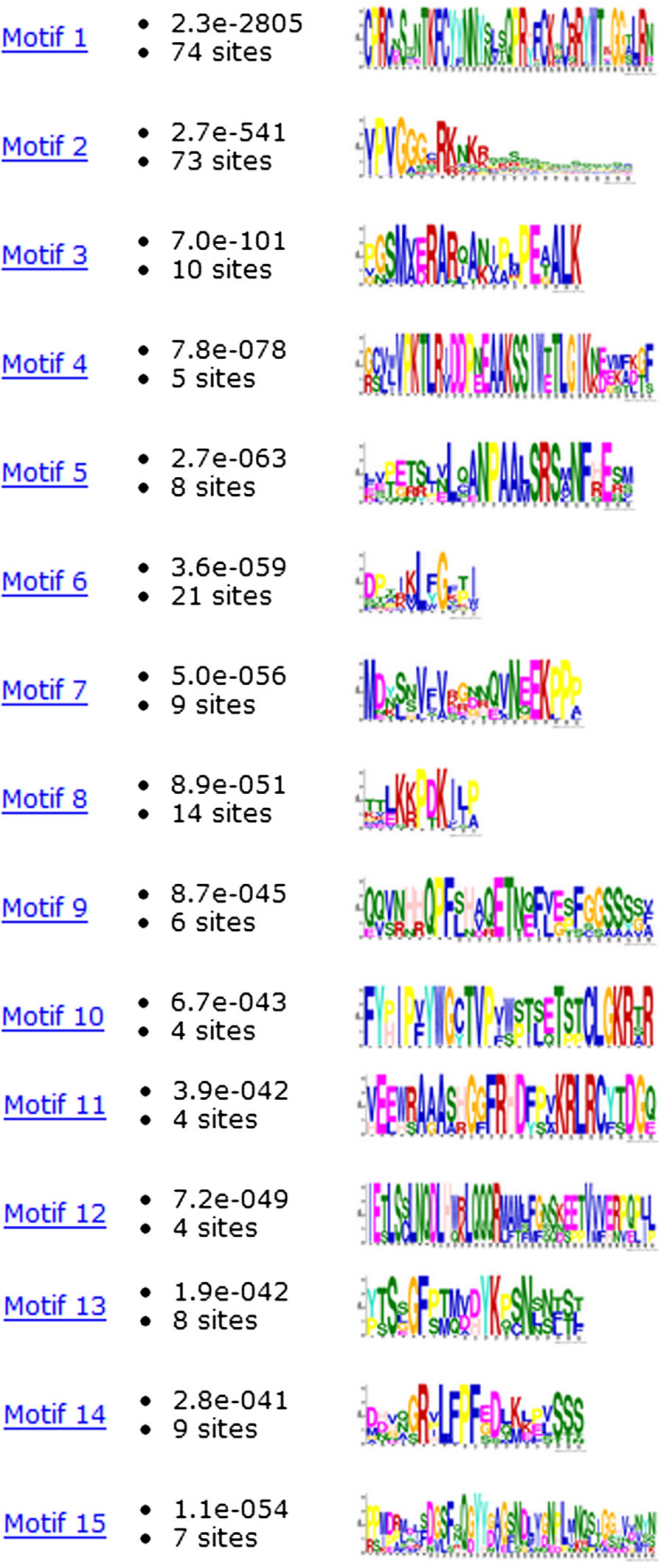
**Sequence logos of Dof domains in Chinese cabbage.** The overall height of the stack indicates the level of sequence conservation. Heights of residues within a stack indicate the relative frequency of each residue at that position.

Most of the 76 BraDof family proteins in Chinese cabbage classified into nine classes have 15 motifs. A total of 76 BraDof family proteins were analyzed by multiple sequence alignment of their Dof domains. All 76 BraDof family proteins had a very highly conserved zf-Dof domain. MEME Suite was used to identify the motifs of the amino acid sequences of the BraDof proteins, and 15 motifs were identified (Figures 2 and 3). All of the BraDofs contained motifs 1 and 2, which suggests that all BraDofs have a highly conserved domain. Moreover, each class of BraDofs had several special motifs at their C-terminal regions. One BraDof factor of Class A did not have motif 6, and one BraDof factor of Class D_2.1_ did not have motifs 13 and 14. Similar motifs on the C-terminal regions determine the phylogenetic relationship of BraDofs.

**Figure 4:**
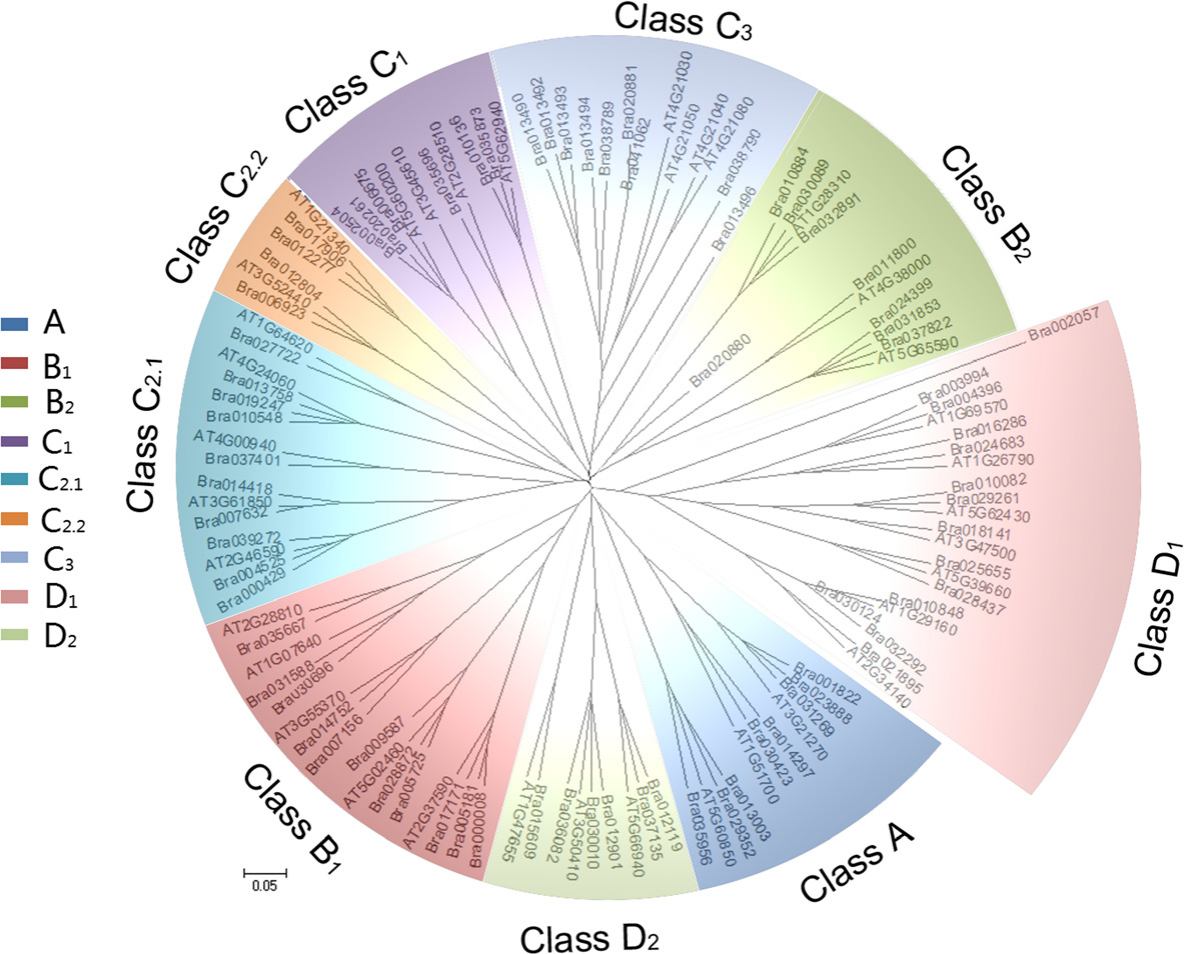
**Unrooted phylogenetic tree of BraDofs in Chinese cabbage.** The amino acid sequences of the DBD were aligned using Clustal W, and the phylogenetic tree was constructed using MEGA 5.

To explain the phylogenetic relationships of the BraDof family transcription factors, a neighbor-joining tree was constructed for phylogenetic analysis to classify the 76 BraDof transcription factors into nine classes, based on the full-length amino acid sequences of BraDofs from Chinese cabbage and AtDofs from *Arabidopsis* [21] (Figure 4). These AtDofs’ amino acid sequences are respectively most similar to BraDofs’ from the same class.

### The evolution of BraDofs transcription factors among plant species

**Figure 5:**
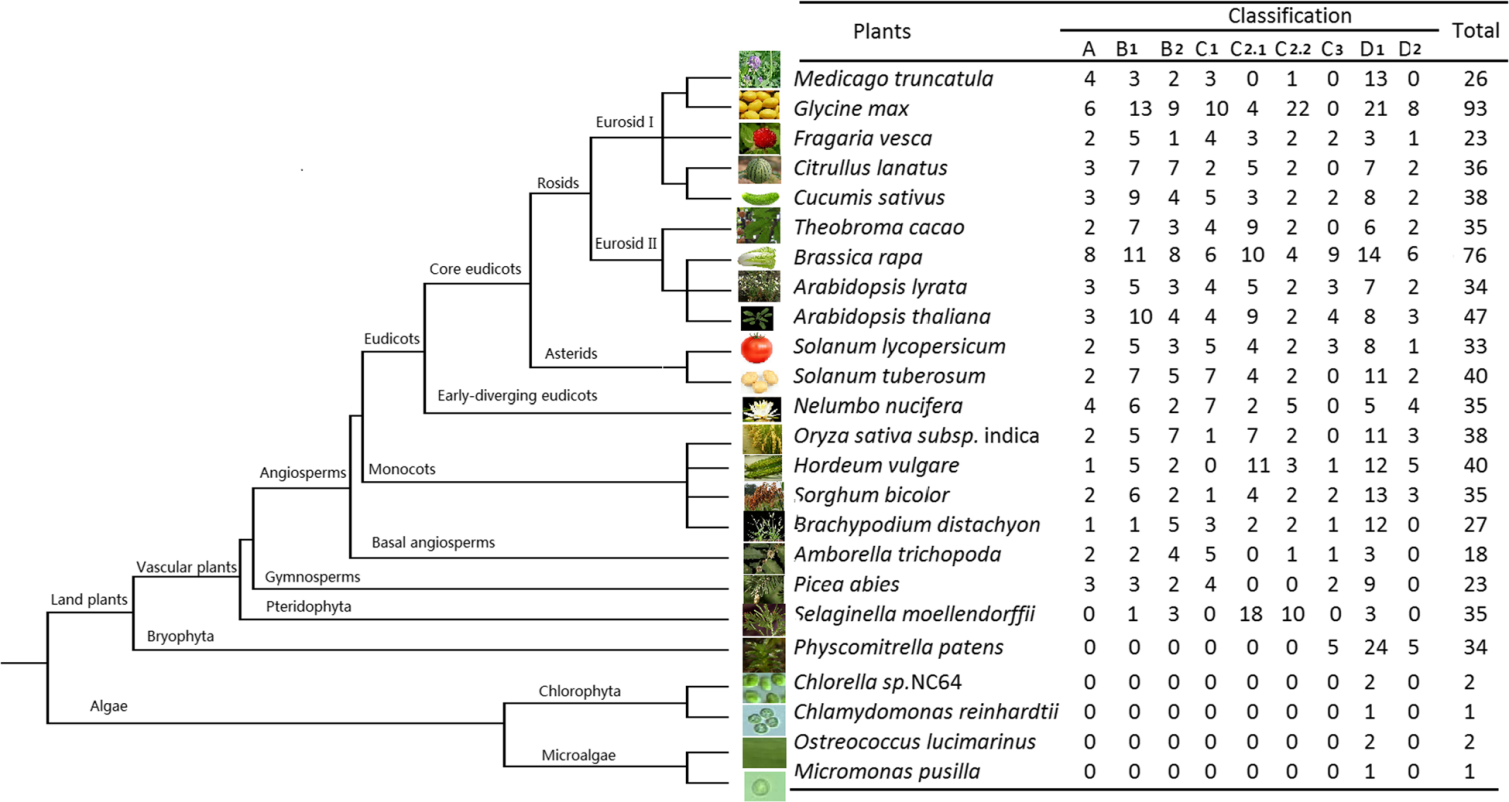
**Comparisons of Dof transcription factors in different species.**

To research the evolution of BraDofs in plant species, comparisons of Dof transcription factors in different plant species were constructed. Compared with other species, Chinese cabbage has a relatively big Dof family, with the 76 Dof members. Except soy and Chinese cabbage, the numbers of Dof members in other higher plants are almost ranged from 30 to 50. Only one or two members were identified in each algae, and there were all belonged to Class D_1_. Moreover, Class D_1_ is also the biggest class in many plants (Figure 5). A total of 76 BraDof family factors were identified in Chinese cabbage, and only 47 AtDof factors was found in *Arabidopsis*. The genome sizes of Chinese cabbage and Arabidopsis were 485 Mb and 125 Mb, respectively. The Dof factors density decreased from 0.376/Mb to 0.157/Mb in Arabidopsis and Chinese cabbage. The results were consistent with the report by Mun et al. [22].

### Location and duplication of *BraDof* genes in Chinese cabbage chromosomes

**Figure 6:**
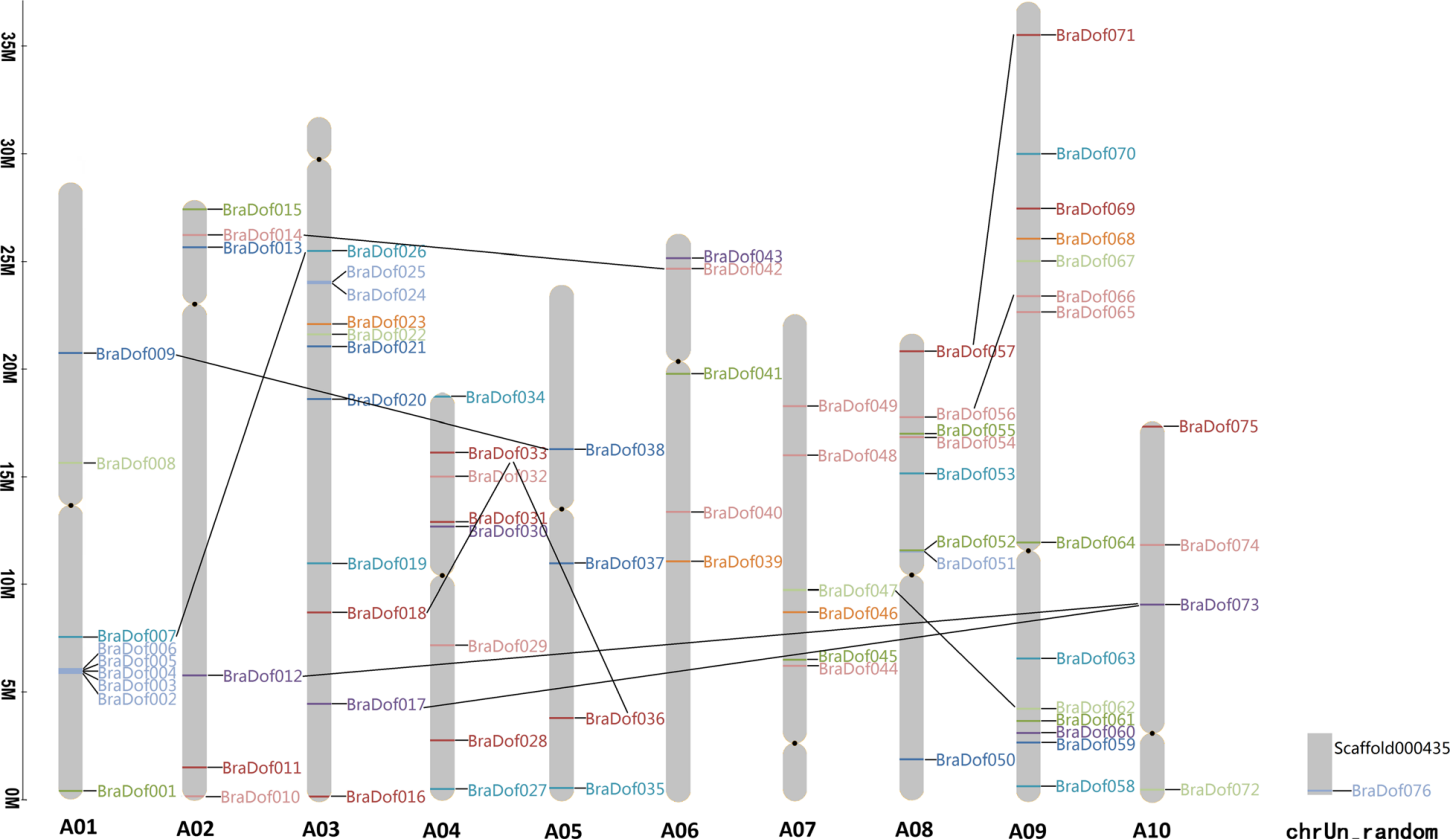
**Chromosomal locations and predicted clusters of** ***BraDof*** **genes in Chinese cabbage.**

To determine the genomic distribution of *BraDof* genes, the DNA sequence of each *BraDof* gene was used to search the Chinese cabbage genome database using BLASTN. Although each of the 10 Chinese cabbage chromosomes contained several *BraDof* genes, their distributions seemed uneven, and a single *BraDof* gene was located on Scaffold 000435 (Figure 6). Fourteen *BraDof* genes were located on chromosome 01; the largest number of *BraDof* genes was observed in this chromosome. Chromosomes 05 and 10 had only 4 *BraDof* genes each, showing the fewest *BraDof* genes among all of the chromosomes studied. Chromosomes A04 and A08 had 8 *BraDof* genes. No *BraDof* genes were found on the short arms of chromosomes A03 and A07, but 11 and 6 *BraDofs* were respectively found on their long arms. Several gene clusters, such as *BraDof 002–006* on A01 and *BraDof 025* and *BraDof 026* on A03, were also found on the chromosomes of Chinese cabbage. Interestingly, all of these genes belonged to Class C_3_.

Potential duplicated genes were marked on the 10 chromosomes and Scaffold 000435 according to the synonymous substitution rate and synteny data (Additional file 1: Tables S13 and S14). Ten duplication events were predicted on these chromosomes, and these duplication events occurred in six classes of genes, including Classes A, B_1_, C_1_, C_2.1_, D_1_, and D_2_ (Figure 6). None of the potential duplications were intra chromosomal duplication events, and all of the duplications occurred on two different chromosomes. Furthermore, the duplicate genes belonged to the same classes. Two special genes duplicating with two genes were discovered: *BraDof033*, which was classified in Class B_1_, duplicated with *BraDof018* and *BraDof036*, and *BraDof073*, which belonged to Class C_1_, duplicated with *BraDof012* and *BraDof017*.

**Figure 7:**
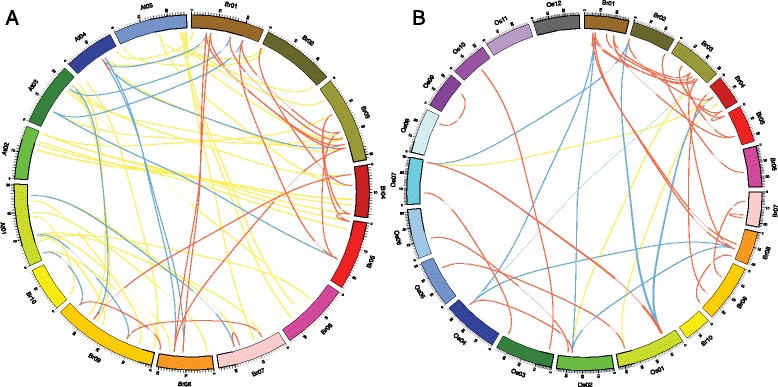
**Gene duplication and synteny analysis of** ***Dof*** **genes between Chinese cabbage and two other model plant species.** Ten Chinese cabbage (A01 to A10), five *Arabidopsis* chromosome (Chr1 to Chr5) and twelve rice (Os1 to Os12) maps were based on the orthologous and paralogous pair positions, and demonstrate highly conserved synteny. The red lines represent the orthologous *Dof* genes between Chinese cabbage and two other model plant species. **(A)** Gene duplication and synteny analysis of *Dof* genes between Chinese cabbage and *Arabidopsis*. The green and blue lines represent the paralogous *Dof* genes in Chinese cabbage and *Arabidopsis*, respectively. Colored lines connecting two chromosomal regions denote syntenic regions of genome. **(B)** Gene duplication and synteny analysis of *Dof* genes between Chinese cabbage and *O. sativa*. The green and blue lines represent the paralogous *Dof* genes in Chinese cabbage and *O. sativa*, respectively. Colored lines connecting two chromosomal regions denote syntenic regions of genome.

Now, the whole genome *A. thaliana* and Chinese cabbage were analyzed very deeply and thoroughly, and found that Chinese cabbage has undergone triplication events since its divergence from *A. thaliana* [23]. Moreover, *A. thaliana* and *O. sativa* are two model plants, which were belonged to dicotyledon and monocotyledon, respectively. Therefore, we constructed two comparative syntenic maps of Chinese cabbage associated with *A. thaliana* and *O. sativa*, respectively (Figure 7). Thirty-seven and seven pairs of orthologous *Dof* genes were found between *BraDofs* and *AtDofs* and between *BraDofs* and *OsDofs*, respectively. And there were nine and thirty paralogs located in genome of *O. sativa* and Chinese cabbage, respectively. In addition, thirteen and ten pairs of corthologous *Dof* genes were investigated in the Chinese cabbage genome and *O. sativa* genome (Figure 7).

### The interaction network of *BraDof* genes

**Figure 8:**
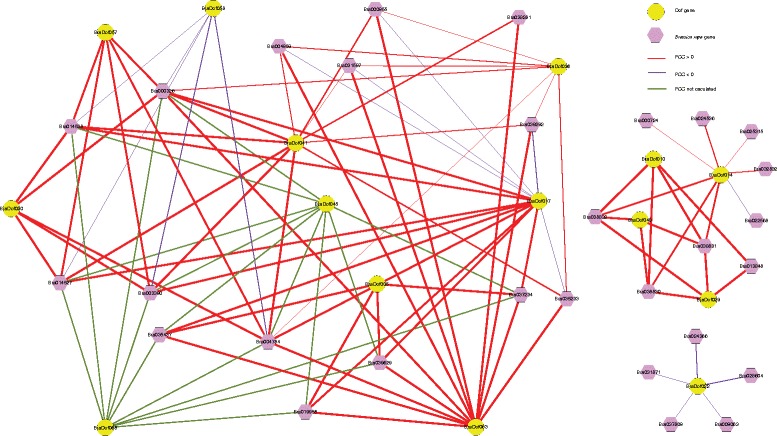
**The interaction networks of Dofs in Chinese cabbage according to the orthologs in** ***Arabidopsis*** **.** The PCC represents the Pearson Correlation Coefficient.

To further research the *BraDof* genes how to interact with other genes, an interaction network associated with *AtDofs Arabidopsis* orthologs was built according to *BraDof* genes from Chinese cabbage (Figure 8). The red, purple and green lines stand for positive correlation (Pearson correlation coefficient >0) with seventy-one pairs of interacting genes, negative correlation (Pearson correlation coefficient <0) with sixteen pairs of interacting genes and unclear correlation (Pearson correlation coefficient not calculated) with sixteen pairs of interacting genes. The interaction network of *BraDofs* showed a very complicated correlation with other genes in Chinese cabbage, which may indicate *BraDof* genes involve in many fundamental mechanisms by regulating many downstream factors or being regulated by many upstream genes.

### Expression analysis of *BraDof* genes under stress treatments in two Chinese cabbage varieties

To further investigate the functions of *BraDof* genes correlated with their expression, 9 *BraDof* genes were subjected to qRT-PCR to examine the expression profiles of *BraDof* genes under cold, heat, salt, and drought treatments in ‘Lubaisanhao’ and ‘Qingdao 87-114’. The 9 *BraDof* genes were selected from the different 9 subgroups on behalf of these 9 subgroups to do qRT-PCR for expression analysis. The 9 genes were representative and can explain the expression profiles of genes from 9 different classes. Each of these 9 genes having a highly conserved Dof domain should be more similar to their homologous genes, and the efficiency of the primers based on their 3’ terminal sequences are better.

**Figure 9:**
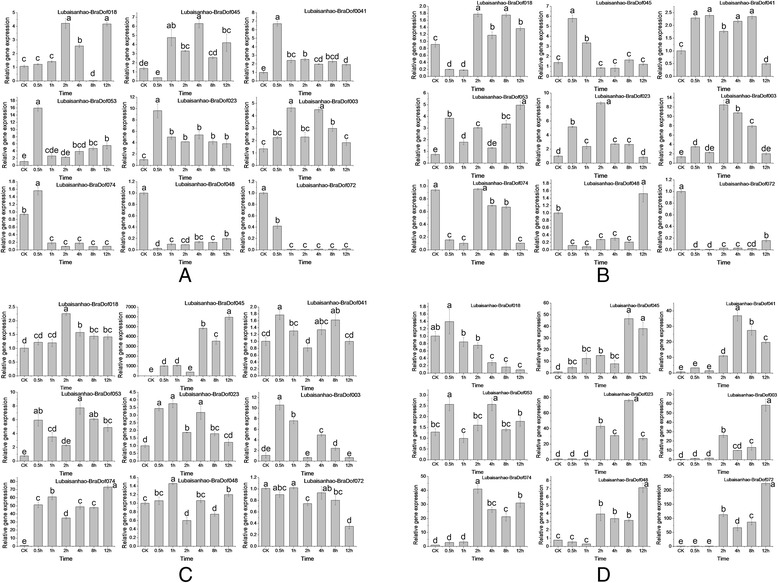
**Expression patterns of** ***BraDof*** **genes in the Chinese cabbage variety ‘Lubaisanhao’ under heat, cold, salt, and drought treatments. (A)**. Expression patterns at 38°C; **(B)**. Expression patterns at 4°C; **(C)**. Expression patterns under salt treatment; **(D)**. Expression patterns under drought treatment. Different letters indicate significant differences between different time treatments stages in the same cultivar (*P* < 0.05).

**Figure 10:**
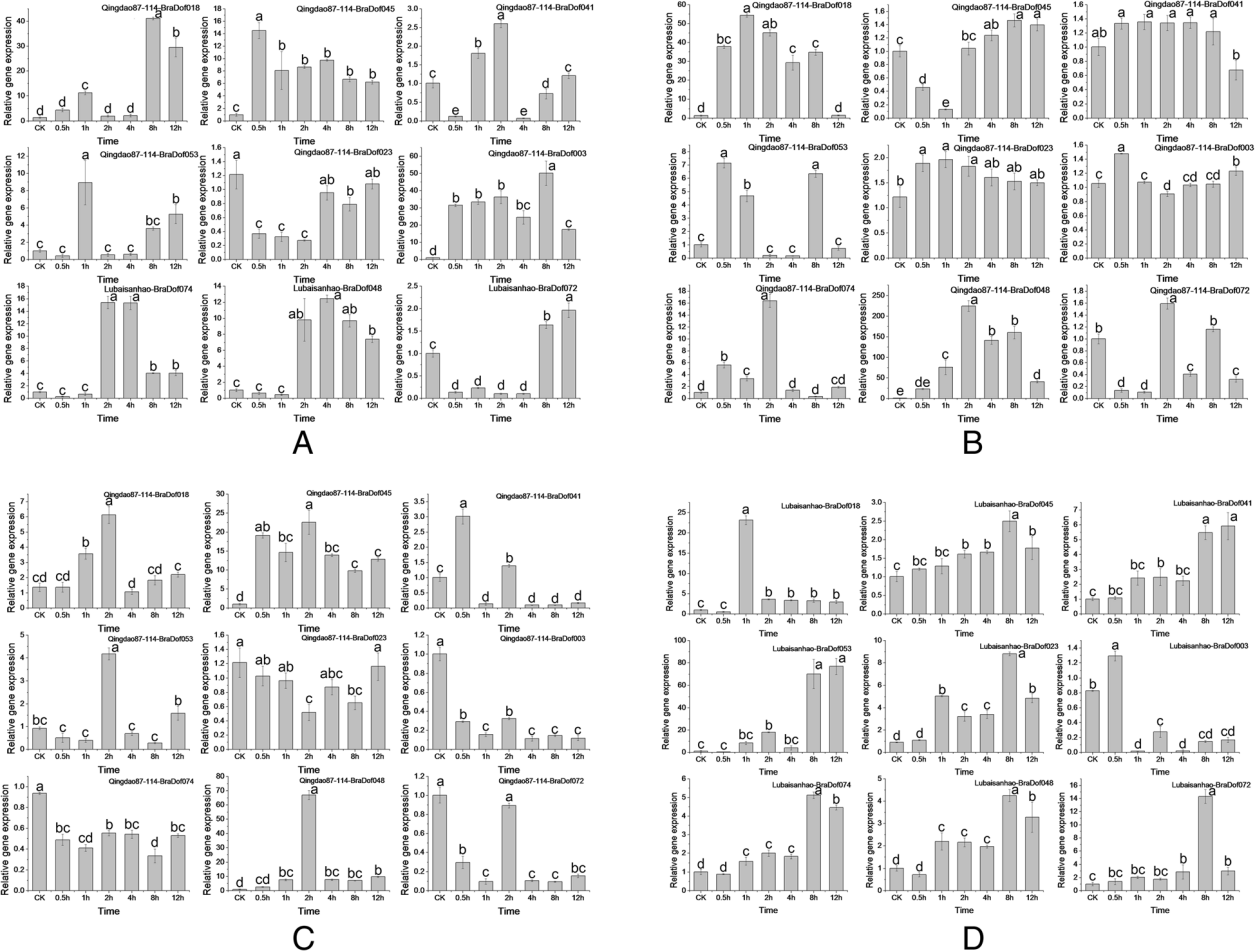
**Expression patterns of** ***BraDof*** **genes in the Chinese cabbage variety ‘Qingdao87-114’ under heat, cold, salt, and drought treatments. (A)**. Expression patterns at 38°C; **(B)**. Expression patterns at 4°C; **(C)**. Expression patterns under salt treatment; **(D)**. Expression patterns under drought treatment. Different letters indicate significant differences between different time treatments stages in the same cultivar (*P* < 0.05).

While most of the *BraDof* genes were up regulated under the four stress treatments (Figures 9 and 10), some notable exceptions were observed. The *BraDof072* gene was down regulated by cold, heat, and drought stress treatments in ‘Lubaisanhao’ and by salt stress treatment in ‘Qingdao87-114’. In addition, *BraDof048* was down regulated in ‘Lubaisanhao’ by heat treatment, and *BraDof003* and *BraDof074* were down regulated in ‘Qingdao87-114’ by salt treatment. *BraDof023* in ‘Qingdao87-114’ was initially down regulated by heat and salt stress treatments and then up regulated after 4 h. The gene of *BraDof074* showed complicated expression. The gene was down regulated in ‘Qingdao87-114’ by salt treatment. However, in ‘Lubaisanhao’, the gene was first down regulated by cold stress treatment and then up regulated later on. Under heat conditions, opposite findings were observed (Figure 9).

## Discussion

### Overall *Dof* gene families in maize, Arabidopsis, barley, wheat and rice

The gene encoding Dof protein, named *ZmDof1*, was first isolated in maize and found to be involved in C4 photosynthesis [24]. A total of 30 *Dof* family genes in rice, 36 genes in *Arabidopsis*, 24 genes in barley, 31 genes in wheat, and 54 genes in maize have been identified and analyzed since then [21,25-27]. In higher plants, Dof family transcription factors play important roles in many biological and physiological processes regulating growth, development, defense mechanisms, and flowering in plants. In *Arabidopsis*, AtDAG1, a phloem-specific transcription factor, is involved in light-quality responses during photomorphogenesis; AtCOG1 and OBF3 function in the same manner [16,28-30]. Another Dof factor in *Arabidopsis*, cycling DOF factor 1, principally negatively mediates the expression of *CONSTANS* to control the photoperiodic pathway of flowering time; CDF2 and CDF3 also show this function [13,31]. Overexpression of *OsDof12* in transgenic rice promotes early flowering [32], and the *OsDof3* gene has been identified to participate in the gibberellin-regulated expression of target genes in rice [33,34]. Several barley Dof family transcription factors, including BPBF (HvDof24), SAD (HvDof23), HvDof 17, and HvDof19, were involved in the regulation of hormonal balance between gibberellin and abscisic acid during seed germination [35-37]. Similar to other zinc fingers, the Dof domain, as a bi-functional domain, regulates both DNA-binding and protein-protein interactions with other regulatory proteins including basic leucine zipper (bZIP), myeloblastosis (MYB) transcription factors and nuclear high mobility group (HMG) proteins [10,13,19,33,35,38-41].

### Genome evolution of the Dof family transcription factors in plant

Dof transcription factors were widely identified in many higher plants [10,28,30,35,37]. With further study, some *Dof* genes were also found in low plants, such as, algaes and moss [25]. A comparison of *Dof* genes from algaes to higher plants in our study showed that higher plants have far more *Dof* genes than algaes, which indicating a huge expansion of Dof family transcription factor members occurred in the evolution process of low plants to land plants. Furthermore, only class D_1_ members exist in algaes inferred that the other Dof classes’ members may originate from class D_1_ members.

The genome of Chinese cabbage has undergone triplication events since its divergence from *A. thaliana* [23]. The genome size of Chinese cabbage (485 Mb) is more than three times larger than that of *Arabidopsis* (125 Mb), while the number of BraDof transcription factors (76 members) is only less than twice of *Arabidopsis* (47 members). This outcome may result from alternative actions during the triplicate evolution. The number of Dof members varies among land plants [20], potato contains a great amount of family members of Dof transcription factors (93 members), notably more than that of lower plants, such as in algae [10,14,42], only one Dof factor was found in the green alga *C. reinhardtii* and no identifiable Dof factor in the red alga *Cyanidioschyzon merolae*. This result demonstrated that the origin of the Dof transcription factors pre-dates the divergence of the green algae and the ancestors of terrestrial plants [25,42]. Dof transcription factors are associated with the evolution of plants [42,43].

### Gene duplication of *BraDof* genes in Chinese cabbage

It is well known that gene or genome duplication events are the primary sources of genetic novelty [43-45]. Gene duplication was the main effect in gene family expansion, the more *Dof* genes emerged in higher plants indicated the domain duplication was occurred in eukaryotic evolution [43]. Novel *Dof* genes arise through divergence of duplicated genes after either single gene duplication, segmental duplication, or whole-genome duplication from aglae to angiosperm [23,46]. The triplication event between Chinese cabbage and *A. thaliana* has revealed that *A. thaliana* as a main ancestral constituting the current diploid Chinese cabbage. The distribution of the *Dof* family genes in the whole Chinese cabbage genome likely resulted from segment and whole-genome duplication [23,43]. A diagram of the chromosomal position of *BraDof* genes was constructed to determine the distribution of *BraDof* genes in Chinese cabbage. Although each of the 10 Chinese cabbage chromosomes contained several *BraDof* genes and a single *BraDof* gene, *BraDof076*, was located on Scaffold 000435, the distributions of the genes were uneven. Genome duplication has occurred throughout plant evolution [47]. Ten pairs of potential duplicate genes were found in Chinese cabbage chromosomes. Interestingly, each pair of duplicate genes belonged to the same class. Two special genes had duplicate relationships with two other genes: *BraDof033* duplicated with *BraDof018* and *BraDof036* and *BraDof073* duplicated with *BraDof012* and *BraDof017*. Several *BraDof* genes were clustered on chromosomes 01 and 03 of Chinese cabbage, and these clustered genes were all classified in Class C_3_. These results indicate that the expansion of *BraDof* genes in Chinese cabbage chromosomes is related to the network and duplication events of *BraDof* genes of Chinese cabbage. The uneven distribution of*BraDof* gene locations on the chromosomes maybe related to the genome duplication of Chinese cabbage.

Moreover, an amount of *BraDof-AtDof* and *BraDof-OsDof* gene pairs were identified in several pairs of duplicated genomic patterns. The number of orthologous events of *BraDof-AtDof* genes is more than the number of *BraDof-OsDof* genes. Furthermore, pairwise orthologs were found between *Arabidopsis* and Chinese cabbage (Figure 7). During the evolution of Chinese cabbage from *Arabidopsis*, triplication events had occurred by whole-genome analysis [23,44]. For this reason, this result may indicate *BraDof* genes in Chinese cabbage share the similar structure and function with *AtDof* genes in *Arabidopsis*. Higher plants have developed complex response mechanisms of gene regulation network and pathway to stress resistance mechanisms, which include biochemical, physiological, cellular and molecular processes [7,48]. When plants are exposed to various abiotic stresses, thousands of transcription factors, including Dof family genes, are activated to adjust the physiological and biochemical pathways [49,50].

### The interaction of Dof transcription factors with other factors

Dof proteins have been shown to interact with specific basic leucine zipper (bZIP) proteins, nuclear high mobility group (HMG) proteins, and other Dof proteins [38-40]. Most of the BraDof positively or negatively interact with a wide range of Protein kinase C (PKC) (Figure 8). Furthermore, according to NCBI blast, BraDof 022 was potentially involved in negatively interacting with five other bZIPs (Figure 8), which is similar with previous studies. Some other BraDofs associated with some tolerant factors, such as TPr (translocated promoter region) protein, TCP (named after the first three characterized members: *teosinte-branched 1* (*tb1*) from *Zea mays* [51], *CYCLOIDEA* (*CYC*) from *Antirrhinum majus* [52] and *PROLIFERATION CELL FACTOR 1* and *2* (*PCF1* and *PCF2*) from *Oryza sativa* [53]) proteins, and so on. Protein kinase C isozymes transduce a massive number of extracellular signals causing generation of the lipid second messenger diacylglycerol (DAG), therefore regulating diverse cellular behaviors, such as survival, growth and proliferation, migration, and apoptosis; consequently, their dysregulation is associated with a plethora of pathophysiologies [54,55]. Moreover, nucleoporin Tpr is a component of the nuclear pore complex (NPC) that localizes exclusively to intranuclear filaments. Tpr functions as a scaffolding element in the nuclear phase of the NPC and plays a role in mitotic spindle checkpoint signaling [56,57]. These genes positively or negatively regulated by BraDofs were involved in abiotic stress tolerance more or less. The BraDofs mediate other factors might be a complicated pathway to respond abiotic stress in Chinese cabbage.

### Dof transcription factors response abiotic stress in Chinese cabbage

The Dof family of transcription factors is a large group of factors that are found mainly in plants. In plant, Dof family transcription factors play very important roles in development as well as in hormonal regulation and stress responses. However, little is known about this family in Chinese cabbage [10,14,25,37]. In particular, the abiotic stress response mechanisms of *BraDof* genes in Chinese cabbage are unknown [58]. Understanding BraDofs functions in Chinese cabbage will help to develop characterize of Chinese cabbage cultivars such as salt and drought tolerance. The expression profiles analysis of large numbers of *BraDof* genes provides a powerful tool for identifying groups of the *BraDof* genes and discovering novel regulators involved in the signal transport of abiotic stress responses in Chinese cabbage. The mechanism of the BraDofs’ responses to the four abiotic stresses (cold, heat, high salinity, and drought) also needs further investigation.

The expression profiles of nine selected *BraDof* genes from nine classes under four stress treatments (salt, drought, heat, and cold) were investigated using qRT-PCR in the Chinese cabbage varieties ‘Qingdao87-114’ and ‘Lubaisanhao’. Most genes showed up-regulated expression profiles under these four stresses in both varieties (Figures 9 and 10). Moreover, up-regulated expressions of these genes were always observed about 2 h after stress treatments, which indicate that *BraDof* gene expression increases immediately under application of abiotic stresses in Chinese cabbage.

As revealed by qRT-PCR expression profiles analysis, all *BraDof* genes respond to different abiotic stresses, high salinity, drought, and extreme temperatures with different timing in leaves, indicating that these genes might participate in abiotic stress responses. Corrales and his colleagues demostrated that transgenic *Arabidpsis* hosting the *SlCDF3* gene (a tomato Dof factor) exhibited higher expression levels of abitioc stress-responsive genes under non-stress conditions, like *COP15*, *RD29A* and *ERD10*, which suggested that *SlCDFs* might function as up-stream regulators in drought and salt stress response pathways [58]. Most of the *BraDof* genes were up-regulated quickly (before 2 h) by the four stress treatments, although notable exceptions may be observed. Some genes were down-regulated, and the up-regulated expression was not simply up regulated. The results indicated that the defense actions of *BraDof* genes respond quickly to induce other *BraDof* family genes, and they also might cause the down regulation of gene expression in activation of plant resistance. Our study demonstrates that BraDof transcription factors play a crucial role in plant resistance to abiotic stresses *via* a complicated reaction mechanism. With the accumulation or reduction of *BraDof* genes expression levels, the interacting factors may be regulated to accumulating or reducing for abiotic tolerance. The results provide a better understanding of the functions of *BraDof* genes in Chinese cabbage.

## Conclusions

We performed a comprehensive analysis of Dof family factors in Chinese cabbage. A total of 76 genes encoding BraDof family transcription factor were identified from Chinese cabbage, and those BraDof factors were divided into nine classes: Classes A, B_1_, B_2_, C_1_, C_2.1_, C_2.2_, C_3_, D_1_ and D_2_. The proportion of each class of BraDof factors was calculated based on phylogenetic relationships. Ten duplicate events of *BraDof* genes were discovered in Chinese cabbage chromosomes. The uneven distribution of *BraDof* genes in *Brassica* chromosomes may cause the expansion of *BraDof* genes. Most of the selected *BraDof* genes were induced by the four abiotic stresses (cold, heat, salt and drought) treatments. In addition, the comparative study of the Dof family factors between Chinese cabbage and other plant species provided valuable information for further function studies of the Dof factors in Chinese cabbage. Our study will support recourses for understanding how BraDof transcription factors respond to abiotic stresses and the interaction of these genes for abiotic stress response mechanism.

## Methods

### Database searches and analysis for BraDof family members in Chinese cabbage

The nucleotide and protein sequences of BraDof factors from *B. rapa* L. ssp. *pekinensis* were collected from the BRAD (http://brassicadb.org/brad/) database [23]. We used the BLAST program available on the NCBI (National Center for Biotechnology Information) database to search for potential Dof domains (http://www.ncbi.nlm.nih.gov/). The sequences of all Dof transcription factors in other species were downloaded from the plant TFDB database (http://planttfdb.cbi.edu.cn/) [59].

### BraDof domain, motif identification, and phylogenetic analysis

MEME Suite was used to identify all motifs in the BraDof protein sequences [60]. Analysis was performed using the following parameters: number of repetitions, if any; optimum width of the motif, 6–50; and maximum number of motifs, 15. Identified BraDof domains were aligned using ClustalW with default parameters. A phylogenetic tree was constructed using MEGA 5.0, comparing 47 *Arabidopsis thaliana* Dof domains using the neighbor-joining method with a bootstrap value of 1,000 [61,62].

### Putative protein characteristic prediction and duplication analysis of BraDofs

Several physiological and biochemical indices were also analyzed. The theoretical pI was calculated by ExPASy (http://web.expasy.org/compute_pi/) [63]. Sequence Manipulation Suite (http://www.bio-soft.net/sms/) was used to analyze protein statistics, and the solubility of recombinant proteins was predicted by the RPSP program (http://biotech.ou.edu) [64]. The synteny of the *BraDof* genes was calculated by MicroSyn software according to Cai’s means (http://fcsb.njau.edu.cn/microsyn/) [65].

### Identification of orthologous and paralogous genes

OrthoMCL was used to search for orthologous and paralogous genes in Chinese cabbage, *Arabidopsis*, and rice using the entire Dof protein sequences [66]. We used an E-value of 1e^−5^ for BLASTP and OrthoMCL analyses, gathered the orthologous and paralogous relationships, and displayed them using the Circos software (http://circos.ca/) [67]. An interaction network of *BraDof* genes from Chinese cabbage was constructed to understand the genome-wide regulation network. An interaction work of *Arabidopsis* Dof proteins was constructed using the *Arabidopsis* Interactions Viewer (http://bar.utoronto.ca/interactions/cgi-bin/arabidopsis_interactions_viewer.cgi). By replacing *Arabidopsis* proteins with corresponding orthologous and co-orthologous Chinese cabbage proteins, an interaction network of BraDof proteins from Chinese cabbage was constructed and displayed using the Cytoscape software (http://www.cytoscape.org/).

### Plant materials, growth conditions and stress treatments

Seeds of ‘Lubaisanhao’ and ‘Qingdao87-114’ were obtained from the State Key Laboratory of Crop Genetics and Germplasm Enhancement in Nanjing Agricultural University. The seeds were germinated at 25°C for 2 d and grown in a growth chamber with a photoperiod of 14 h of light and 10 h of darkness at 28°C. Young leaves were harvested for RNA isolation. For temperature stress treatments, whole four-leaf seedlings were exposed to 4°C or 38°C. For salt stress treatments, four-leaf seedlings were incubated with 100 mM NaCl. For drought stress treatments, four-leaf seedlings were incubated with 15% PEG 6000. Leaves were harvested 0, 0.5, 1, 2, 4, 8, and 12 h after application of the stress treatments, immediately frozen in liquid nitrogen, and then stored at −70°C for RNA isolation.

### Total RNA isolation and cDNA synthesis

Total RNA was isolated from plant materials using a total RNA kit (Tiangen, Beijing, China) according to the manufacturer’s protocol. A total of 1 μg of RNA of each sample was used to synthesize first strand cDNA using M-MLV reverse transcriptase according to the manufacturer’s instructions (TaKaRa, Dalian, China). cDNA was diluted 20-fold for quantitative real-time PCR (qRT-PCR) analysis.

### qRT-PCR analysis

To analyze *BraDof* gene transcription levels under abiotic stress, we performed qRT-PCR on an Applied Biosystems 7500 real-time PCR system using a SYBR Green RT-PCR kit (Novland, Shanghai, China). The PCR thermal cycle conditions were as follows: denaturation at 95°C for 30 s, 40 cycles of 95°C for 10 s, and 58°C for 20 s. At the end of each cycle, fluorescence intensities were measured for qRT-PCR. A melting curve (61 cycles at 65°C for 10 s) was generated to check for specific amplification. The method of calculating relative gene expression was performed as described by Pfaffl using the 2^−ΔΔCq^ method [68]. Briefly, the expression profiles were performed with modifications as follows: Ratio = 2^−ΔΔCq^; ΔΔCq = ΔCq (sample) − ΔCq (control); ΔC_q_ = C_q_(target) − C_q_(reference). The experiments were repeated three times for different abiotic stress treatments. Standard errors of means among replicates were also calculated. A statistical method (*t* test) for significant differences was analyzed using the SPSS statistics software (version 17.0) [69].

The primers used for relative quantification were designed from the coding sequences of BraDof transcription factors in Chinese cabbage; all of the primers used in this experiment are provided in Table 1. All primers used for relative quantification were synthesized by Genscript Nanjing Inc. (Nanjing, China).

**Table 1:** **Primer sequences used for qRT-PCR amplification of** ***actin*** **and 9** ***Dof*** **genes**

**Name**	**Oligonucleotides sequences**
Actin-Forward primer	AGTGGGCGTACTACTGGTATTGTG
Actin-Reverse primer	AGAGAATCAGTGAGGTCCCTACCC
Bra030423F1-Forward primer	CCCGACCAGAAACCTAAC
Bra030423R1-Reverse primer	CGTAACTAATCCCGACATCC
Bra014752F1-Forward primer	ATCTTCAAATGCGTTGTATC
Bra014752R1-Reverse primer	ACTCCGTTCCCATCCTTA
Bra030089F1-Forward primer	CTATTTGGGTCTTCGGTAT
Bra030089R1-Reverse primer	ATTGACAACACTCGCATT
Bra035873F1-Forward primer	TGGGAGTGAACAATAACAA
Bra035873R1-Reverse primer	CCAATCAAGCGATAACAG
Bra010548F1-Forward primer	TGCCAGTTTCTTCGTCTT
Bra010548R1-Reverse primer	TCCTCCACTCAGCATCCC
Bra012804F1-Forward primer	ATGGGTTGCCTCCGTTTC
Bra012804R1-Reverse primer	GCTCCAGCTTCCGAACAA
Bra013492F1-Forward primer	CAAGGTTATTACGGTGCT
Bra013492R1-Reverse primer	TGCCGACATTATGGTTCA
Bra002057F1-Forward primer	CTGGTGGAAAGATGAGGT
Bra002057R1-Reverse primer	CATTGATTCGCAGAGGAC
Bra012119F1-Forward primer	ACAACGGTGATGTTACGGC
Bra012119R1-Reverse primer	CAAGTCCAAACCCTTCCAG

## Additional file

Additional file 1: Table S1. C2C2-Dof-like genes in Chinese cabbage chromosome 1. **Table S2.** C2C2-Dof-like genes in Chinese cabbage chromosome 2. **Table S3.** C2C2-Dof-like genes in Chinese cabbage chromosome 3. **Table S4.** C2C2-Dof-like genes in Chinese cabbage chromosome 4. **Table S5.** C2C2-Dof-like genes in Chinese cabbage chromosome 5. **Table S6.** C2C2-Dof-like genes in Chinese cabbage chromosome 6. **Table S7.** C2C2-Dof-like genes in Chinese cabbage chromosome. **Table S8**. C2C2-Dof-like genes in Chinese cabbage chromosome 8. **Table S9.** C2C2-Dof-like genes in Chinese cabbage chromosome 9. **Table S10.** C2C2-Dof-like genes in Chinese cabbage chromosome 10. **Table S11.** C2C2-Dof-like genes in Chinese cabbage scaffold. **Table S12.** Theoreticalvalues of Dofs in Chinese cabbage. **Table S13.** *K*_s_ values of potentially duplicated genes in Chinese cabbage. **Table S14.** Number of homologies of potentially duplicated genes in Chinese cabbage.
