# Supplementary material for: Genome-wide analysis of Dof family transcription factors and their responses to abiotic stresses in Chinese cabbage
Source: BMC Genomics. 2015 Jan 31;16(1):33. doi: 10.1186/s12864-015-1242-9 (PMC4320540; doi:10.1186/s12864-015-1242-9)
Supplement: Additional file 1: Table S1. — C2C2-Dof-like genes in Chinese cabbage chromosome 1. Table S2. C2C2-Dof-like genes in Chinese cabbage chromosome 2. Table S3. C2C2-Dof-like genes in Chinese cabbage chromosome 3. Table S4. C2C2-Dof-like genes in Chinese cabbage chromosome 4. Table S5. C2C2-Dof-like genes in Chinese cabbage chromosome 5. Table S6. C2C2-Dof-like genes in Chinese cabbage chromosome 6. Table S7. C2C2-Dof-like genes in Chinese cabbage chromosome. Table S8. C2C2-Dof-like genes in Chinese cabbage chromosome 8. Table S9. C2C2-Dof-like genes in Chinese cabbage chromosome 9. Table S10. C2C2-Dof-like genes in Chinese cabbage chromosome 10. Table S11. C2C2-Dof-like genes in Chinese cabbage scaffold. Table S12. Theoreticalvalues of Dofs in Chinese cabbage. Table S13. K s values of potentially duplicated genes in Chinese cabbage. Table S14. Number of homologies of potentially duplicated genes in Chinese cabbage. [file 12864_2015_1242_MOESM1_ESM.docx]

**Additional file**

**Additional file 1: Table S1.** C2C2-Dof-like genes in Chinese cabbage chromosome 1

| NO. | Name | Sub-group | Gene name | Chromosome | Start | Stop |
| --- | --- | --- | --- | --- | --- | --- |
|  | BraDof001 | Class B_2_ | [Bra011800](javascript:modalDialog('multiSearch.php?gene=Bra011800','select%20database',390,200)) | A01 | 446168 | 446896 |
|  | BraDof002 | Class C_3_ | [Bra013490](javascript:modalDialog('multiSearch.php?gene=Bra013490','select%20database',390,200)) | A01 | 5948821 | 5949558 |
|  | BraDof003 | Class C_3_ | [Bra013492](javascript:modalDialog('multiSearch.php?gene=Bra013492','select%20database',390,200)) | A01 | 5961676 | 5962401 |
|  | BraDof004 | Class C_3_ | [Bra013493](javascript:modalDialog('multiSearch.php?gene=Bra013493','select%20database',390,200)) | A01 | 5967996 | 5968748 |
|  | BraDof005 | Class C_3_ | [Bra013494](javascript:modalDialog('multiSearch.php?gene=Bra013494','select%20database',390,200)) | A01 | 5971382 | 5972053 |
|  | BraDof006 | Class C_3_ | [Bra013496](javascript:modalDialog('multiSearch.php?gene=Bra013496','select%20database',390,200)) | A01 | 5978264 | 5978557 |
|  | BraDof007 | Class C_2.1_ | [Bra013758](javascript:modalDialog('multiSearch.php?gene=Bra013758','select%20database',390,200)) | A01 | 7625855 | 7626865 |
|  | BraDof008 | Class D_2_ | [Bra030010](javascript:modalDialog('multiSearch.php?gene=Bra030010','select%20database',390,200)) | A01 | 15732296 | 15733003 |
|  | BraDof009 | Class A | [Bra023888](javascript:modalDialog('multiSearch.php?gene=Bra023888','select%20database',390,200)) | A01 | 20831090 | 20831692 |

**Additional file 1: Table S2.** C2C2-Dof-like genes in Chinese cabbage chromosome 2

| NO. | Name | Sub-group | Gene name | Chromosome | Start | Stop |
| --- | --- | --- | --- | --- | --- | --- |
|  | BraDof010 | Class D_1_ | [Bra028437](javascript:modalDialog('multiSearch.php?gene=Bra028437','select%20database',390,200)) | A02 | 312740 | 314235 |
|  | BraDof011 | Class B_1_ | [Bra028872](javascript:modalDialog('multiSearch.php?gene=Bra028872','select%20database',390,200)) | A02 | 1672058 | 1673491 |
|  | BraDof012 | Class C_1_ | [Bra020261](javascript:modalDialog('multiSearch.php?gene=Bra020261','select%20database',390,200)) | A02 | 5902800 | 5903516 |
|  | BraDof013 | Class A | [Bra029352](javascript:modalDialog('multiSearch.php?gene=Bra029352','select%20database',390,200)) | A02 | 25698254 | 25699090 |
|  | BraDof014 | Class D_1_ | [Bra029261](javascript:modalDialog('multiSearch.php?gene=Bra029261','select%20database',390,200)) | A02 | 26259413 | 26260935 |
|  | BraDof015 | Class B_2_ | [Bra031853](javascript:modalDialog('multiSearch.php?gene=Bra031853','select%20database',390,200)) | A02 | 27457284 | 27458198 |

**Additional file 1: Table S3.** C2C2-Dof-like genes in Chinese cabbage chromosome 3

| NO. | Name | Sub-group | Gene name | Chromosome | Start | Stop |
| --- | --- | --- | --- | --- | --- | --- |
|  | BraDof016 | Class B_1_ | [Bra005725](javascript:modalDialog('multiSearch.php?gene=Bra005725','select%20database',390,200)) | A03 | 303585 | 305064 |
|  | BraDof017 | Class C_1_ | [Bra006675](javascript:modalDialog('multiSearch.php?gene=Bra006675','select%20database',390,200)) | A03 | 4567529 | 4569812 |
|  | BraDof018 | Class B_1_ | [Bra000008](javascript:modalDialog('multiSearch.php?gene=Bra000008','select%20database',390,200)) | A03 | 8809708 | 8810896 |
|  | BraDof019 | Class C_2.1_ | [Bra000429](javascript:modalDialog('multiSearch.php?gene=Bra000429','select%20database',390,200)) | A03 | 11062602 | 11064429 |
|  | BraDof020 | Class A | [Bra001822](javascript:modalDialog('multiSearch.php?gene=Bra001822','select%20database',390,200)) | A03 | 18666665 | 18667282 |
|  | BraDof021 | Class A | [Bra013003](javascript:modalDialog('multiSearch.php?gene=Bra013003','select%20database',390,200)) | A03 | 21091147 | 21092031 |
|  | BraDof022 | Class D_2_ | [Bra012901](javascript:modalDialog('multiSearch.php?gene=Bra012901','select%20database',390,200)) | A03 | 21630236 | 21630964 |
|  | BraDof023 | Class C_2.2_ | [Bra012804](javascript:modalDialog('multiSearch.php?gene=Bra012804','select%20database',390,200)) | A03 | 22138538 | 22139263 |
|  | BraDof024 | Class C_3_ | [Bra038790](javascript:modalDialog('multiSearch.php?gene=Bra038790','select%20database',390,200)) | A03 | 24031315 | 24031761 |
|  | BraDof025 | Class C_3_ | [Bra038789](javascript:modalDialog('multiSearch.php?gene=Bra038789','select%20database',390,200)) | A03 | 24034783 | 24035403 |
|  | BraDof026 | Class C_2.1_ | [Bra019247](javascript:modalDialog('multiSearch.php?gene=Bra019247','select%20database',390,200)) | A03 | 25483969 | 25485396 |

**Additional file 1: Table S4.** C2C2-Dof-like genes in Chinese cabbage chromosome 4

| NO. | Name | Sub-group | Gene name | Chromosome | Start | Stop |
| --- | --- | --- | --- | --- | --- | --- |
|  | BraDof027 | Class C_2.1_ | [Bra014418](javascript:modalDialog('multiSearch.php?gene=Bra014418','select%20database',390,200)) | A04 | 504454 | 505386 |
|  | BraDof028 | Class B_1_ | [Bra014752](javascript:modalDialog('multiSearch.php?gene=Bra014752','select%20database',390,200)) | A04 | 2790018 | 2791643 |
|  | BraDof029 | Class D_1_ | [Bra025655](javascript:modalDialog('multiSearch.php?gene=Bra025655','select%20database',390,200)) | A04 | 7217292 | 7219102 |
|  | BraDof030 | Class C_1_ | [Bra035696](javascript:modalDialog('multiSearch.php?gene=Bra035696','select%20database',390,200)) | A04 | 12760498 | 12761268 |
|  | BraDof031 | Class B_1_ | [Bra035667](javascript:modalDialog('multiSearch.php?gene=Bra035667','select%20database',390,200)) | A04 | 12969106 | 12970498 |
|  | BraDof032 | Class D_1_ | [Bra021895](javascript:modalDialog('multiSearch.php?gene=Bra021895','select%20database',390,200)) | A04 | 15086238 | 15086744 |
|  | BraDof033 | Class B_1_ | [Bra017171](javascript:modalDialog('multiSearch.php?gene=Bra017171','select%20database',390,200)) | A04 | 16214577 | 16215840 |
|  | BraDof034 | Class C_2.1_ | [Bra039272](javascript:modalDialog('multiSearch.php?gene=Bra039272','select%20database',390,200)) | A04 | 18793965 | 18795047 |

**Additional file 1: Table S5.** C2C2-Dof-like genes in Chinese cabbage chromosome 5

| NO. | Name | Sub-group | Gene name | Chromosome | Start | Stop |
| --- | --- | --- | --- | --- | --- | --- |
|  | BraDof035 | Class C_2.1_ | [Bra004525](javascript:modalDialog('multiSearch.php?gene=Bra004525','select%20database',390,200)) | A05 | 617912 | 618970 |
|  | BraDof036 | Class B_1_ | [Bra005181](javascript:modalDialog('multiSearch.php?gene=Bra005181','select%20database',390,200)) | A05 | 3872685 | 3873844 |
|  | BraDof037 | Class A | [Bra030423](javascript:modalDialog('multiSearch.php?gene=Bra030423','select%20database',390,200)) | A05 | 11118178 | 11118735 |
|  | BraDof038 | Class A | [Bra031269](javascript:modalDialog('multiSearch.php?gene=Bra031269','select%20database',390,200)) | A05 | 16470027 | 16470602 |

**Additional file 1: Table S6.** C2C2-Dof-like genes in Chinese cabbage chromosome 6

| NO. | Name | Sub-group | Gene name | Chromosome | Start | Stop |
| --- | --- | --- | --- | --- | --- | --- |
|  | BraDof039 | Class C_2.2_ | [Bra017906](javascript:modalDialog('multiSearch.php?gene=Bra017906','select%20database',390,200)) | A06 | 8781267 | 8782028 |
|  | BraDof040 | Class D_1_ | [Bra018141](javascript:modalDialog('multiSearch.php?gene=Bra018141','select%20database',390,200)) | A06 | 10565337 | 10567116 |
|  | BraDof041 | Class B_2_ | [Bra024399](javascript:modalDialog('multiSearch.php?gene=Bra024399','select%20database',390,200)) | A06 | 15509564 | 15510484 |
|  | BraDof042 | Class D_1_ | [Bra010082](javascript:modalDialog('multiSearch.php?gene=Bra010082','select%20database',390,200)) | A06 | 19262121 | 19263467 |
|  | BraDof043 | Class C_1_ | [Bra010136](javascript:modalDialog('multiSearch.php?gene=Bra010136','select%20database',390,200)) | A06 | 19651530 | 19653036 |

**Additional file 1: Table S7**. C2C2-Dof-like genes in Chinese cabbage chromosome 7

| NO. | Name | Sub-group | Gene name | Chromosome | Start | Stop |
| --- | --- | --- | --- | --- | --- | --- |
|  | BraDof044 | Class D_1_ | [Bra030124](javascript:modalDialog('multiSearch.php?gene=Bra030124','select%20database',390,200)) | A07 | 6383816 | 6384184 |
|  | BraDof045 | Class B_2_ | [Bra030089](javascript:modalDialog('multiSearch.php?gene=Bra030089','select%20database',390,200)) | A07 | 6663630 | 6664526 |
|  | BraDof046 | Class C_2.2_ | [Bra012277](javascript:modalDialog('multiSearch.php?gene=Bra012277','select%20database',390,200)) | A07 | 8878653 | 8879438 |
|  | BraDof047 | Class D_2_ | [Bra012119](javascript:modalDialog('multiSearch.php?gene=Bra012119','select%20database',390,200)) | A07 | 9906519 | 9907181 |
|  | BraDof048 | Class D_1_ | [Bra003994](javascript:modalDialog('multiSearch.php?gene=Bra003994','select%20database',390,200)) | A07 | 16169873 | 16171586 |
|  | BraDof049 | Class D_1_ | [Bra004396](javascript:modalDialog('multiSearch.php?gene=Bra004396','select%20database',390,200)) | A07 | 18431078 | 18432364 |

**Additional file 1: Table S8**. C2C2-Dof-like genes in Chinese cabbage chromosome 8

| NO. | Name | Sub-group | Gene name | Chromosome | Start | Stop |
| --- | --- | --- | --- | --- | --- | --- |
|  | BraDof050 | Class A | [Bra014297](javascript:modalDialog('multiSearch.php?gene=Bra014297','select%20database',390,200)) | A08 | 1851972 | 1852574 |
|  | BraDof051 | Class C_3_ | [Bra020881](javascript:modalDialog('multiSearch.php?gene=Bra020881','select%20database',390,200)) | A08 | 11473336 | 11474172 |
|  | BraDof052 | Class B_2_ | [Bra020880](javascript:modalDialog('multiSearch.php?gene=Bra020880','select%20database',390,200)) | A08 | 11478606 | 11478839 |
|  | BraDof053 | Class C_2.1_ | [Bra010548](javascript:modalDialog('multiSearch.php?gene=Bra010548','select%20database',390,200)) | A08 | 15098363 | 15099817 |
|  | BraDof054 | Class D_1_ | [Bra010848](javascript:modalDialog('multiSearch.php?gene=Bra010848','select%20database',390,200)) | A08 | 16779855 | 16780382 |
|  | BraDof055 | Class B_2_ | [Bra010884](javascript:modalDialog('multiSearch.php?gene=Bra010884','select%20database',390,200)) | A08 | 16949809 | 16950696 |
|  | BraDof056 | Class D_1_ | [Bra016286](javascript:modalDialog('multiSearch.php?gene=Bra016286','select%20database',390,200)) | A08 | 17710452 | 17711793 |
|  | BraDof057 | Class B_1_ | [Bra030696](javascript:modalDialog('multiSearch.php?gene=Bra030696','select%20database',390,200)) | A08 | 20768848 | 20770123 |

**Additional file 1: Table S9**. C2C2-Dof-like genes in Chinese cabbage chromosome 9

| NO. | Name | Sub-group | Gene name | Chromosome | Start | Stop |
| --- | --- | --- | --- | --- | --- | --- |
|  | BraDof058 | Class C_2.1_ | [Bra037401](javascript:modalDialog('multiSearch.php?gene=Bra037401','select%20database',390,200)) | A09 | 709199 | 710095 |
|  | BraDof059 | Class A | [Bra035956](javascript:modalDialog('multiSearch.php?gene=Bra035956','select%20database',390,200)) | A09 | 2739093 | 2739896 |
|  | BraDof060 | Class C_1_ | [Bra035873](javascript:modalDialog('multiSearch.php?gene=Bra035873','select%20database',390,200)) | A09 | 3199016 | 3200505 |
|  | BraDof061 | Class B_2_ | [Bra037822](javascript:modalDialog('multiSearch.php?gene=Bra037822','select%20database',390,200)) | A09 | 3759280 | 3760134 |
|  | BraDof062 | Class D_2_ | [Bra037135](javascript:modalDialog('multiSearch.php?gene=Bra037135','select%20database',390,200)) | A09 | 4309328 | 4309966 |
|  | BraDof063 | Class C_2.1_ | [Bra027722](javascript:modalDialog('multiSearch.php?gene=Bra027722','select%20database',390,200)) | A09 | 6648185 | 6649159 |
|  | BraDof064 | Class B_2_ | [Bra032891](javascript:modalDialog('multiSearch.php?gene=Bra032891','select%20database',390,200)) | A09 | 12053056 | 12053949 |
|  | BraDof065 | Class D_1_ | [Bra032292](javascript:modalDialog('multiSearch.php?gene=Bra032292','select%20database',390,200)) | A09 | 22804911 | 22805438 |
|  | BraDof066 | Class D_1_ | [Bra024683](javascript:modalDialog('multiSearch.php?gene=Bra024683','select%20database',390,200)) | A09 | 23536369 | 23537570 |
|  | BraDof067 | Class D_2_ | [Bra036082](javascript:modalDialog('multiSearch.php?gene=Bra036082','select%20database',390,200)) | A09 | 25175005 | 25175673 |
|  | BraDof068 | Class C_2.2_ | [Bra006923](javascript:modalDialog('multiSearch.php?gene=Bra006923','select%20database',390,200)) | A09 | 26199994 | 26200722 |
|  | BraDof069 | Class B_1_ | [Bra007156](javascript:modalDialog('multiSearch.php?gene=Bra007156','select%20database',390,200)) | A09 | 27619255 | 27620238 |
|  | BraDof070 | Class C_2.1_ | [Bra007632](javascript:modalDialog('multiSearch.php?gene=Bra007632','select%20database',390,200)) | A09 | 30159100 | 30167067 |
|  | BraDof071 | Class B_1_ | [Bra031588](javascript:modalDialog('multiSearch.php?gene=Bra031588','select%20database',390,200)) | A09 | 35686154 | 35687368 |

**Additional file 1: Table S10.** C2C2-Dof-like genes in Chinese cabbage chromosome 10

| NO. | Name | Sub-group | Gene name | Chromosome | Start | Stop |
| --- | --- | --- | --- | --- | --- | --- |
|  | BraDof072 | Class D_2_ | [Bra015609](javascript:modalDialog('multiSearch.php?gene=Bra015609','select%20database',390,200)) | A10 | 654945 | 655616 |
|  | BraDof073 | Class C_1_ | [Bra002504](javascript:modalDialog('multiSearch.php?gene=Bra002504','select%20database',390,200)) | A10 | 9181265 | 9182169 |
|  | BraDof074 | Class D_1_ | [Bra002057](javascript:modalDialog('multiSearch.php?gene=Bra002057','select%20database',390,200)) | A10 | 11878503 | 11883500 |
|  | BraDof075 | Class B_1_ | [Bra009587](javascript:modalDialog('multiSearch.php?gene=Bra009587','select%20database',390,200)) | A10 | 17328895 | 17329682 |

**Additional file 1: Table S11**. C2C2-Dof-like genes in Chinese cabbage scaffold

| NO. | Name | Sub-group | Gene name | Chromosome | Start | Stop |
| --- | --- | --- | --- | --- | --- | --- |
|  | BraDof076 | Class C_3_ | [Bra041062](javascript:modalDialog('multiSearch.php?gene=Bra041062','select%20database',390,200)) | Scaffold000435 | 10500 | 11012 |

**Additional file 1: Table S12.** Theoreticalvalues of Dofs in Chinese cabbage

| Gene name | Size (aa) | MW (Da) | pI | Percentage of aliphatic amino acids /% | Percentage of aromatics amino acids /% | Percentage of positive amino acids /% | Percentage of negative amino acids /% | Recombinant protein solubility  (percent of insolubility)  /% |
| --- | --- | --- | --- | --- | --- | --- | --- | --- |
| BraDof001 | 244 | 26994.30 | 9.68 | 13 | 9 | 13 | 6 | 97.9 |
| BraDof002 | 247 | 27477.38 | 9.53 | 14 | 11 | 13 | 6 | >97.0 |
| BraDof003 | 243 | 27566.52 | 9.20 | 15 | 9 | 15 | 7 | >97.0 |
| BraDof004 | 252 | 28349.41 | 8.81 | 14 | 10 | 15 | 8 | >97.0 |
| BraDof005 | 225 | 24682.06 | 6.49 | 14 | 9 | 12 | 9 | 96.0 |
| BraDof006 | 99 | 11483.13 | 9.75 | 16 | 12 | 20 | 6 | 60.1 |
| BraDof007 | 338 | 36290.65 | 8.39 | 12 | 7 | 13 | 8 | >97.0 |
| BraDof008 | 237 | 24599.21 | 9.15 | 12 | 7 | 13 | 8 | >97.0 |
| BraDof009 | 202 | 21518.90 | 9.36 | 10 | 8 | 12 | 8 | >97.0 |
| BraDof010 | 402 | 44028.27 | 6.76 | 14 | 7 | 15 | 13 | 91.9 |
| BraDof011 | 408 | 42843.87 | 9.26 | 13 | 7 | 10 | 4 | >97.0 |
| BraDof012 | 240 | 25692.54 | 8.83 | 11 | 9 | 12 | 8 | >97.0 |
| BraDof013 | 280 | 29970.59 | 7.07 | 11 | 9 | 12 | 9 | 96.9 |
| BraDof014 | 294 | 33406.60 | 8.83 | 15 | 8 | 16 | 12 | 95.1 |
| BraDof015 | 306 | 34175.83 | 8.79 | 14 | 8 | 13 | 8 | 97.7 |
| BraDof016 | 405 | 42483.62 | 9.17 | 13 | 7 | 10 | 4 | >97.0 |
| BraDof017 | 279 | 30181.57 | 8.75 | 11 | 9 | 13 | 8 | 97.9 |
| BraDof018 | 295 | 31349.27 | 9.11 | 13 | 9 | 10 | 5 | >97.0 |
| BraDof019 | 371 | 40532.52 | 8.25 | 13 | 7 | 12 | 7 | >97.0 |
| BraDof020 | 207 | 22294.91 | 9.15 | 9 | 7 | 13 | 8 | >97.0 |
| BraDof021 | 296 | 31439.34 | 7.07 | 11 | 9 | 11 | 9 | 97.3 |
| BraDof022 | 244 | 25436.02 | 8.13 | 13 | 9 | 10 | 7 | 97.5 |
| BraDof023 | 243 | 26416.93 | 5.40 | 15 | 10 | 12 | 12 | 84.1 |
| BraDof024 | 150 | 17026.02 | 9.74 | 12 | 9 | 18 | 9 | 87.0 |
| BraDof025 | 208 | 23306.59 | 6.65 | 15 | 9 | 14 | 11 | 96.4 |
| BraDof026 | 345 | 37272.90 | 7.65 | 12 | 7 | 13 | 8 | 97.9 |
| BraDof027 | 312 | 34384.73 | 9.28 | 10 | 8 | 12 | 6 | >97.0 |
| BraDof028 | 352 | 38434.91 | 8.77 | 12 | 9 | 9 | 7 | >97.0 |
| BraDof029 | 447 | 47707.87 | 5.42 | 12 | 6 | 12 | 13 | 90.6 |
| BraDof030 | 258 | 27917.90 | 8.69 | 14 | 10 | 11 | 8 | >97.0 |
| BraDof031 | 346 | 36922.57 | 9.25 | 13 | 6 | 11 | 4 | >97.0 |
| BraDof032 | 170 | 18775.45 | 9.48 | 17 | 7 | 20 | 10 | 76.2 |
| BraDof033 | 311 | 33118.10 | 9.24 | 12 | 7 | 9 | 5 | >97.0 |
| BraDof034 | 362 | 39236.04 | 9.06 | 13 | 6 | 11 | 6 | >97.0 |
| BraDof035 | 354 | 38487.43 | 9.30 | 14 | 6 | 12 | 6 | >97.0 |
| BraDof036 | 327 | 34858.10 | 9.06 | 13 | 7 | 11 | 8 | >97.0 |
| BraDof037 | 187 | 20879.38 | 8.94 | 13 | 8 | 15 | 10 | 97.2 |
| BraDof038 | 193 | 21052.45 | 9.34 | 9 | 8 | 14 | 8 | 97.9 |
| BraDof039 | 255 | 28547.27 | 4.77 | 13 | 11 | 11 | 14 | 54.6 |
| BraDof040 | 429 | 46956.37 | 8.85 | 12 | 7 | 13 | 10 | 97.3 |
| BraDof041 | 308 | 33936.49 | 8.85 | 13 | 8 | 12 | 8 | 97.9 |
| BraDof042 | 270 | 30227.02 | 8.57 | 16 | 8 | 16 | 11 | 94.0 |
| BraDof043 | 370 | 39390.51 | 7.67 | 14 | 7 | 13 | 7 | >97.0 |
| BraDof044 | 124 | 13614.57 | 9.59 | 13 | 11 | 19 | 8 | 57.0 |
| BraDof045 | 300 | 32185.78 | 9.55 | 15 | 7 | 13 | 4 | >97.0 |
| BraDof046 | 263 | 29157.03 | 4.98 | 14 | 10 | 11 | 13 | 72.5 |
| BraDof047 | 222 | 23276.72 | 9.10 | 13 | 11 | 11 | 6 | >97.0 |
| BraDof048 | 388 | 42620.07 | 9.00 | 12 | 9 | 13 | 9 | 97.9 |
| BraDof049 | 370 | 40967.41 | 8.55 | 13 | 8 | 13 | 9 | 97.8 |
| BraDof050 | 202 | 21984.44 | 9.26 | 13 | 7 | 13 | 8 | 97.9 |
| BraDof051 | 280 | 31721.64 | 8.15 | 13 | 11 | 15 | 9 | 97.3 |
| BraDof052 | 79 | 8763.10 | 10.14 | 16 | 8 | 21 | 3 | 55.6 |
| BraDof053 | 354 | 38403.19 | 8.35 | 13 | 7 | 14 | 8 | 97.9 |
| BraDof054 | 177 | 19427.06 | 9.48 | 15 | 9 | 18 | 9 | 61.6 |
| BraDof055 | 297 | 31864.35 | 9.17 | 17 | 7 | 12 | 4 | >97.0 |
| BraDof056 | 337 | 37956.81 | 7.62 | 14 | 8 | 16 | 12 | 93.7 |
| BraDof057 | 336 | 35882.61 | 9.49 | 15 | 7 | 12 | 4 | >97.0 |
| BraDof058 | 300 | 33473.59 | 7.75 | 10 | 8 | 15 | 9 | 97.2 |
| BraDof059 | 269 | 28646.37 | 7.13 | 20 | 9 | 13 | 9 | 96.4 |
| BraDof060 | 342 | 36648.60 | 8.05 | 14 | 7 | 13 | 8 | 97.9 |
| BraDof061 | 286 | 31748.23 | 9.09 | 13 | 9 | 13 | 7 | >97.0 |
| BraDof062 | 214 | 22573.04 | 9.20 | 13 | 12 | 11 | 7 | 97.9 |
| BraDof063 | 326 | 34620.11 | 8.46 | 13 | 8 | 12 | 8 | >97.0 |
| BraDof064 | 299 | 32341.92 | 9.26 | 14 | 7 | 13 | 5 | >97.0 |
| BraDof065 | 177 | 19276.75 | 9.35 | 13 | 8 | 17 | 9 | 82.2 |
| BraDof066 | 329 | 37133.90 | 7.00 | 13 | 9 | 13 | 11 | 91.9 |
| BraDof067 | 224 | 23903.51 | 8.80 | 14 | 9 | 10 | 6 | >97.0 |
| BraDof068 | 244 | 26843.62 | 6.04 | 15 | 10 | 14 | 11 | 87.9 |
| BraDof069 | 329 | 35696.45 | 9.31 | 14 | 9 | 11 | 7 | >97.0 |
| BraDof070 | 1057 | 118826.21 | 8.29 | 17 | 8 | 14 | 11 | 89.0 |
| BraDof071 | 319 | 34605.11 | 9.38 | 14 | 7 | 13 | 5 | >97.0 |
| BraDof072 | 225 | 23337.63 | 6.58 | 14 | 9 | 8 | 8 | >97.0 |
| BraDof073 | 260 | 27834.80 | 8.89 | 10 | 9 | 12 | 7 | >97.0 |
| BraDof074 | 284 | 32280.98 | 9.24 | 22 | 10 | 17 | 9 | 65.5 |
| BraDof075 | 179 | 19327.80 | 9.71 | 14 | 9 | 13 | 3 | 97.4 |
| BraDof076 | 172 | 19420.55 | 8.83 | 14 | 12 | 16 | 9 | 96.9 |

**Additional file 1: Table S13.** *K*_s_ values of potentially duplicated genes in Chinese cabbage

| Genomic_x | Genomic_y | *Ks* |
| --- | --- | --- |
| BraDof007 | BraDof026 | 0.38 |
| BraDof012 | BraDof073 | 0.39 |
| BraDof018 | BraDof033 | 0.38 |
| BraDof047 | BraDof062 | 0.48 |
| BraDof057 | BraDof071 | 0.43 |
| BraDof056 | BraDof066 | 0.37 |
| BraDof017 | BraDof073 | 0.45 |
| BraDof033 | BraDof036 | 0.36 |
| BraDof009 | BraDof038 | 0.34 |
| BraDof014 | BraDof042 | 0.39 |

**Additional file 1: Table S14.** Number of homologies of potentially duplicated genes in Chinese cabbage

| Segment 1 | Segment 2 | Number of homology | Probability |
| --- | --- | --- | --- |
| BraDof007 | BraDof026 | 41 | 7.46276645345449E-68 |
| BraDof012 | BraDof073 | 40 | 7.16674651101173E-63 |
| BraDof018 | BraDof033 | 39 | 4.46992945138832E-62 |
| BraDof047 | BraDof062 | 36 | 1.50966089843684E-56 |
| BraDof057 | BraDof071 | 36 | 1.16891870283298E-60 |
| BraDof056 | BraDof066 | 35 | 1.04513043277177E-54 |
| BraDof017 | BraDof073 | 34 | 9.51539708314295E-51 |
| BraDof033 | BraDof036 | 34 | 2.48305708876264E-54 |
| BraDof009 | BraDof038 | 31 | 3.28744616627928E-46 |
| BraDof014 | BraDof042 | 31 | 6.54918507797817E-55 |
